# Respiratory function in Becker muscular dystrophy: a comprehensive longitudinal study

**DOI:** 10.1136/jnnp-2025-337953

**Published:** 2026-02-27

**Authors:** Pietro Riguzzi, Emma Grover, Marianela Schiava, Meredith K James, Jordi Diaz-Manera, Giorgio Tasca, John Bourke, Holly Borland, Dionne Moat, Maha Elseed, Jassi Michell-Sodhi, Carla Bolaño-Díaz, Karen Wong, Ariele Barreto Haagsma, Emma Robinson, Tara Reeves, Peter Waldock, Elizabeth Harris, Michelle McCallum, Giulio Gadaleta, Elena Pegoraro, Luca Bello, Ben Messer, Robert Muni-Lofra, Volker Straub, Chiara Marini Bettolo, Michela Guglieri

**Affiliations:** 1 Translational and Clinical Research Institute, Newcastle University and Newcastle Hospitals NHS Foundation Trust, John Walton Muscular Dystrophy Research Centre, Newcastle upon Tyne, UK; 2 Department of Neurosciences DNS, Universita degli Studi di Padova, Padua, Italy; 3 Department of Neuroscience "Rita Levi Montalcini”, University of Turin, Torino, Italy; 4 North East Assisted Ventilation Service, Newcastle Upon Tyne Hospitals NHS Foundation Trust, Newcastle upon Tyne, UK

**Keywords:** MUSCULAR DYSTROPHY, NEUROMUSCULAR, RESPIRATORY MEDICINE, DYSTROPHIN, MYOPATHY

## Abstract

**Background:**

Becker muscular dystrophy (BMD) is a rare X-linked neuromuscular disorder predominantly affecting males. While respiratory function has been characterised in small cohorts over limited time frames, comprehensive long-term longitudinal data remain lacking, as do established care guidelines to inform respiratory surveillance in BMD.

**Methods:**

In this retrospective analysis, we present a large longitudinal analysis of respiratory function in patients with BMD, based on 1360 spirometry measurements from a single-centre cohort of 152 patients. Analysed assessments spanned paediatric to adult ages (3.4–86.3 years), with a mean follow-up duration of 11 years per patient.

Linear mixed-effects models were used to study longitudinal changes in respiratory function.

**Results:**

Respiratory decline in BMD appears gradual and variable, typically not affecting children (<18 years of age).

Respiratory support was infrequent (11.2%), always non-invasive and limited to nocturnal use.

Loss of ambulation emerged as a strong predictor of faster decline in forced vital capacity percentage of predicted (FVC%) (estimate −0.58%/year, p=0.002), with the requirement for assistive walking devices marking a critical transitional stage.

Upper limb function, assessed via the PUL 2.0 entry item, correlated significantly with FVC%, particularly among non-ambulant patients (rho=0.60, p=0.02). Cardiac involvement showed a limited effect on respiratory function, likely driven by patients with more advanced cardiomyopathy.

No consistent genotype-phenotype correlations were observed.

**Conclusion:**

These findings provide important evidence to inform clinical management, supporting the recommendation of individualised respiratory monitoring strategies and contributing to the design and interpretation of clinical trials in BMD.

**WHAT IS ALREADY KNOWN ON THIS TOPIC**

- Becker muscular dystrophy (BMD) is an X-linked neuromuscular condition that can be associated with respiratory involvement; however, existing evidence on respiratory function in patients with BMD is limited.

**WHAT THIS STUDY ADDS**

- This large, longitudinal retrospective analysis showed that respiratory decline in BMD is typically gradual, uncommon during childhood and highly variable in adults.
- Loss of ambulation and worsening upper limb function are key predictors of respiratory decline, whereas cardiac involvement was only weakly associated with lower respiratory function; no consistent genotype-phenotype correlations were observed.
- The need for respiratory support in BMD was infrequent and generally limited to nocturnal use.

**HOW THIS STUDY MIGHT AFFECT RESEARCH, PRACTICE OR POLICY**

- These findings provide an evidence base to inform clinical surveillance strategies and care recommendations, and to support the design and interpretation of clinical trials in BMD.

## Introduction

 Becker muscular dystrophy (BMD) is an X-linked recessive neuromuscular disorder characterised by progressive muscle weakness and wasting.^1^ The pooled prevalence of BMD is 1.53 per 100 000 males.^2^ Unlike the more severe allelic form Duchenne muscular dystrophy (DMD), BMD is typically caused by in-frame variants in the *DMD* gene, allowing for the production of a partially functional dystrophin protein.^3 4^

There is considerable phenotypic variability in BMD, with different skeletal muscle involvement, spanning from asymptomatic elevation of creatine kinase to more severe DMD-like phenotypes.^1 5^ Cardiac involvement is common, affecting approximately 50% of patients,^6^ and can vary in severity, from subtle signs of fibrosis to severe dilated cardiomyopathy and cardiac failure.^7 8^ Some large multicentric studies,^9 10^ alongside smaller single-centre cohorts,^11 12^ have described the natural history of BMD. Severe respiratory impairment and the need for respiratory support appear to be less common in BMD compared with DMD^13^ or some forms of limb-girdle muscular dystrophies.^14^ However, the optimal timing for respiratory monitoring and which clinical factors may predict poorer outcomes are still not well defined. While respiratory dysfunction appears to correlate with progressive decline in motor function,^12^ this association is not consistently reported across studies.^9^ Moreover, despite emerging evidence of strong genotype-phenotype correlations in BMD with respect to disease progression and cardiac involvement,^7 9 10^ respiratory function has not been systematically investigated.

Currently, no internationally agreed-upon standards of care exist for BMD. Recent guidelines recommend aligning respiratory surveillance in BMD with that used in other neuromuscular disorders, particularly DMD.^15^ However, the distinct clinical course and variability in respiratory involvement across the dystrophinopathy spectrum, especially at its milder and more severe extremes,^16^ highlight the need for a data-driven approach to respiratory monitoring in individuals with BMD and milder dystrophinopathy phenotypes.

Furthermore, in light of recently available clinical trials in BMD, thoroughly characterising the natural history of respiratory function has become paramount to inform the interpretation of respiratory parameters both for safety monitoring and as potential efficacy endpoints.

In this study, we report a comprehensive overview of respiratory function trajectories in BMD and investigate predicting factors associated with different patterns over time. We draw on extensive longitudinal retrospective data from the John Walton Muscular Dystrophy Research Centre (JWMDRC) cohort, as previously described in an earlier publication.^6^ Through this longitudinal analysis, we aim to inform improved clinical practice and contribute to the development of evidence-based standards of care for BMD.

## Methodology

We collected longitudinal retrospective respiratory data from a large cohort of paediatric and adult patients with a confirmed diagnosis of BMD followed up at the JWMDRC in Newcastle upon Tyne, UK.^6^ The data were obtained through spirometry assessments conducted during routine clinical care visits.

Collected measures included: age at the time of spirometry testing; forced vital capacity (FVC) in litres (L) and as a percentage of predicted values (FVC%) in the sitting position according to international standards^17^; FVC in the lying position (L and % predicted), when available. Additional respiratory parameters included forced expiratory volume in 1 s (FEV_1_) in L and % of predicted (sitting), as well as peak cough flow (PCF) measured in litres per minute (L/min).

Standardised spirometry was performed by trained specialised physiotherapists using a Microlab Spirometer (ML3500MK8) or Easy on-PC spirometry sensor and EasyOne Connect software (ndd Medizintechnik AG, Switzerland). All spirometry measurements were quality control reported according to the Association for Respiratory Technology & Physiology spirometry standards. When the optimal technique was not achieved, the test was reported as suboptimal and excluded from the analysis.

To ensure consistency across multiple years of data collection, the percentage of predicted values was calculated using the Global Lung Function Initiative calculators (race-neutral approach).^18 19^ Standing height was used for ambulant patients and arm span for the non-ambulant cohort.

In addition, respiratory comorbidities were documented based on clinical records. For patients receiving respiratory support, data on age at initiation, device type, hours per day of use and body mass index (BMI) at the last assessment were extracted.

Disease motor milestones were classified into four categories as previously^6^: fully ambulant, ambulant with limitation, ambulant with aids and non-ambulant (online supplemental materials). Patients were classified as non-ambulant if they required the use of a wheelchair on a full-time basis.

The age at disease motor milestone transitions was also recorded, when available.

Information regarding BMD-related cardiac involvement, as defined in the previous publication^6^ (online supplemental materials), was collected, as well as data describing upper limb (UL) motor function (PUL 2.0 entry item).^20^

Finally, *DMD* variants were recorded to explore genotype-phenotype associations.

### Statistical analysis

Descriptive statistics were used to summarise categorical variables as counts and percentages. Continuous variables were reported as mean or median, SE and range, depending on data distribution.

Longitudinal changes in respiratory function were analysed using linear mixed-effects models with random intercepts and random slopes to account for repeated measurements within individuals.

In a first analysis, we fitted a separate linear mixed-effects model for each variable of interest (single-variable model). The variables analysed included loss of ambulation (LoA, patients who were non-ambulant at the last assessment), disease motor milestones, cardiac involvement, *DMD* gene variant subtype, with deletion of exons 45–47 serving as reference subtype, and respiratory comorbidities.

Each model included the variable, age (at time of the respiratory assessment) and their interaction as fixed effects, with random effects for both the intercept and age.

In a second analysis, we built a combined linear mixed-effects model (multi-variable model). This model included the variables that were significant in the single-variable model, along with age and their interactions with age. Random effects for intercept and age were also included. Some variables, although significant in the single-variable model, were excluded from the multi-variable model due to the low number of patients in the corresponding groups.

Analysis of covariance (ANCOVA) was used to assess group differences in FVC% while controlling for age at the last assessment. Pairwise comparisons were adjusted using Bonferroni-corrected post hoc tests to account for multiple comparisons. Correlations between continuous variables were evaluated using Spearman’s rank correlation coefficient. A two-tailed p value <0.05 was considered statistically significant. In all reported linear mixed models, the p values correspond to the interaction between variable and age.

All analyses and figures were generated using R (V.4.2.3).

## Results

### Demographics

Demographic and clinical characteristics of the cohort are summarised in table 1.

**Table 1:** Demographic and clinical characteristics (FVC% predicted, sitting)

Parameter	Value
Number of patients included	152
Number of FVC evaluations	1360
Number of FVC evaluations <18 years	573
Number of FVC evaluations ≥18 years	787
Mean age at spirometry assessment (±SD, range)	28.1 years (±19, 3.4–86.3)
Mean number of evaluations per patient (±SD, range)	9 (±5.8, 1–24)
Mean years of follow-up per patient (±SD, range)	11 years (±7.4, 0–27.4)
Patients who lost ambulation, N (%)	37/152 (24.3%)
Mean age at loss of ambulation* (±SD, range)	43.2 (±16.9, 11.2–77.6)
Patients with cardiac involvement, N (%)	76/152 (50.0%)
Patients with OSA, N (%)	10/152 (6.6%)
Patients with asthma, N (%)	19/152 (12.5%)
Patients with COPD, N (%)	7/152 (4.6%)
Patients with other severe respiratory comorbidities, N (%)	4/152 (2.6%)
*DMD* gene variants	
Deletion of exons 45–47, N (%)	62/152 (40.8%)
Deletion of exons 45–48, N (%)	20/152 (13.2%)
Deletion of exons 45–53, N (%)	10/152 (6.6%)
‘Mild variant’ group†, N (%)	11/152 (7.2%)
‘Other’ variants†, N (%)	49/152 (32.2%)

* Age at loss of ambulation was available for 35/37 patients.

† Further details on molecular characterisation are reported in online supplemental table 1.

COPD, chronic obstructive pulmonary disease; FVC%, forced vital capacity percentage of predicted; FVC, forced vital capacity; OSA, obstructive sleep apnoea.

Longitudinal retrospective respiratory data were collected from clinical records spanning from January 1996 to February 2025. Of 1408 recorded measurements of sitting FVC% across the cohort, 48 were excluded due to suboptimal technique, resulting in 1360 valid assessments (hereafter, FVC% refers to sitting values unless otherwise specified). These comprised 573 evaluations from paediatric patients (<18 years of age) and 787 from adults (≥18 years). A total of 152 patients were included in the analysis, with a mean of 9 evaluations per patient and an average follow-up duration of 11 years. At the time of their last evaluation, approximately one-quarter of patients had lost ambulation.

The most common variant was deletion of exons 45–47 (40.8%), followed by deletions of exons 45–48 (13.2%) and 45–53 (6.6%). Individuals carrying deletions of exon 48, 45–55, 48–51 or 49–51, previously reported to be associated with a milder phenotype,^6^ were grouped together (7.2%). All remaining variants were grouped under the ‘other’ category (32.2%). Cardiac involvement was identified in 50% of patients. The most prevalent respiratory comorbidity was asthma (12.5%) followed by obstructive sleep apnoea syndrome (OSA) (6.6%), and chronic obstructive pulmonary disease (COPD) (4.6%). Other severe respiratory conditions, including idiopathic pulmonary fibrosis, pneumothorax, bronchiectasis and pulmonary infarction, were rare and affected together only 2.6% of the cohort. Comorbidities could present in isolation or in combination within individual patients.

### Longitudinal changes in FVC%, FEV_1_% and clinical predictors

In the overall cohort, yearly changes in FVC% were not significant (estimate +0.056%/year, SE 0.085, p=0.51).

When stratified by age, a significant increase was observed during the paediatric stage (<18 years; estimate +0.79%/year, SE 0.18, p<0.0001), whereas in adulthood (≥18 years of age) FVC% showed a significant decline (−0.36%/year, SE 0.10, p=0.0005).

In the single-variable model, significant predictors of FVC% decline were: LoA (estimate −0.59%/year, SE 0.18, p=0.001), cardiac involvement (estimate −0.35%/year, SE 0.17, p=0.047), COPD (estimate −1.09%/year, SE 0.36, p=0.003) and other rare respiratory comorbidities (estimate −1.45%/year, SE 0.45, p=0.001). Asthma, OSA and *DMD* gene variant subtypes were not significant. Due to the small number of patients with COPD and other severe respiratory conditions (n=8), these variables were excluded from the multivariable model.

**Figure 1:**
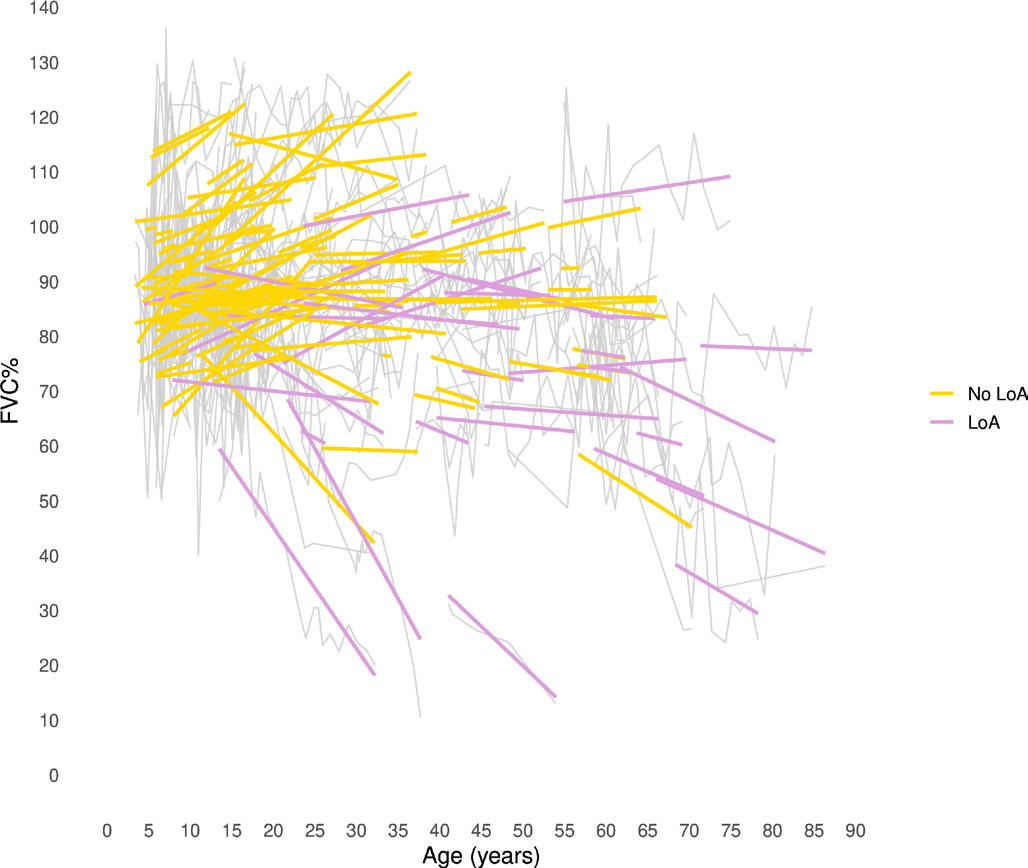
Spaghetti plot showing individual longitudinal trajectories of FVC% raw values (thin grey lines) and model-derived prediction curves for each patient with at least two measurements (coloured lines) from the linear mixed-effects model (multi-variable), stratified by ambulatory status at the last assessment: patients who were non-ambulant (LoA) versus those who remained ambulant. FVC%, forced vital capacity percentage of predicted; LoA, loss of ambulation.

In the multivariable model, LoA remained a significant predictor of FVC% decline (−0.58%/year, SE 0.18, p=0.002; figure 1), whereas cardiac involvement was no longer significant (−0.20%/year, SE 0.17, p=0.25).

A similar analysis was fitted to assess longitudinal changes in FEV_1_% of predicted (1205 measurements across 148 individuals, online supplemental tables 2,3). Interestingly, patients with deletion of exons 45–53 showed a borderline significant positive difference in estimated trajectory (+0.69%/year, SE 0.34, p=0.046) compared with the reference group (deletion of exons 45–47). Similarly to FVC%, in the multivariable model, LoA was a significant predictor for FEV_1_% decline (estimate −0.50%/year, SE 0.19, p=0.008), but cardiac involvement was not significant (estimate −0.24%/year, SE 0.17, p=0.17).

### Group-wise comparison of FVC% across disease motor milestones

**Figure 2:**
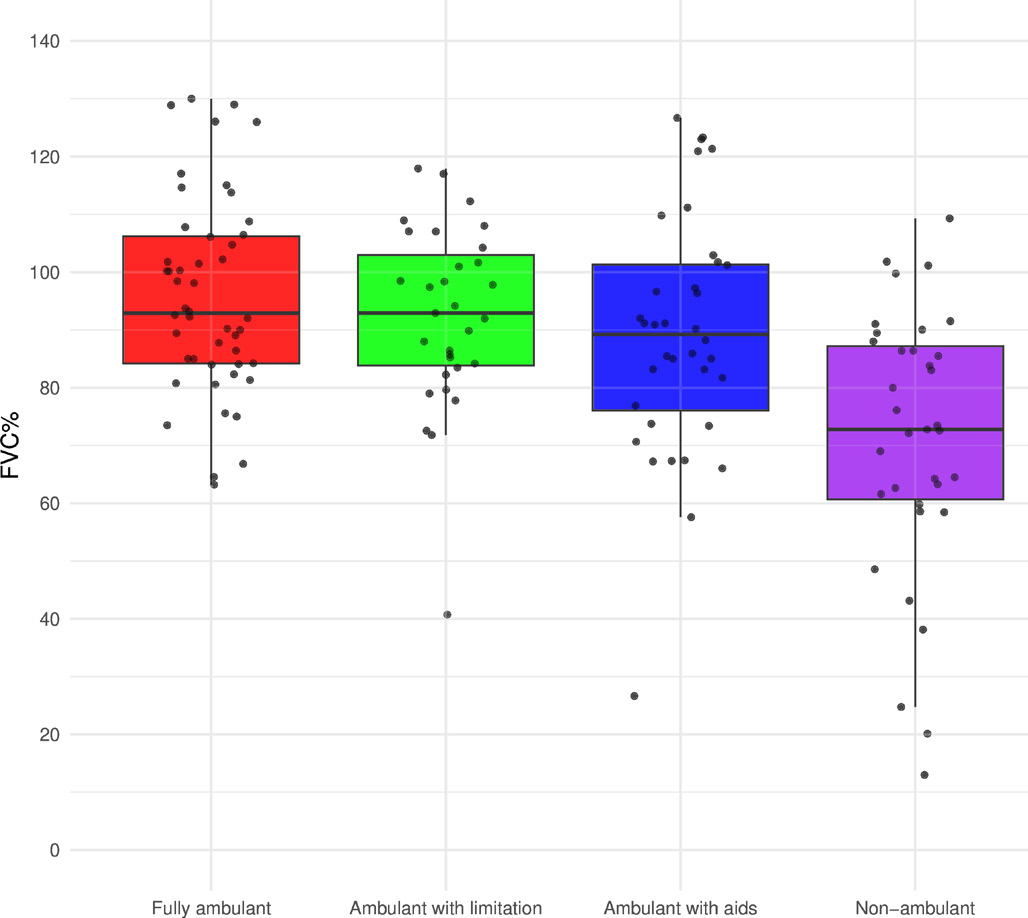
Boxplot (median and IQR) of FVC% distribution at the last assessment by disease motor milestones. Fully ambulant: n=48. Ambulant with limitation: n=31. Ambulant with aids: n=36. Non-ambulant: n=35. FVC%, forced vital capacity percentage of predicted.

Disease motor milestones at the last spirometry assessment could be established for 150 individuals (figure 2). An ANCOVA showed a significant effect of motor milestone on FVC% (p=0.015) after adjusting for age (p=0.004). Post-hoc comparisons with Bonferroni correction revealed that non-ambulant individuals had significantly lower FVC% than all other groups (non-ambulant vs fully ambulant p<0.0001, non-ambulant vs ambulant with limitation p<0.0001, non-ambulant vs ambulant with aids p=0.0007), while differences between other disease motor milestones were not significant.

### FVC% trajectories and predictions by disease motor milestone

**Figure 3:**
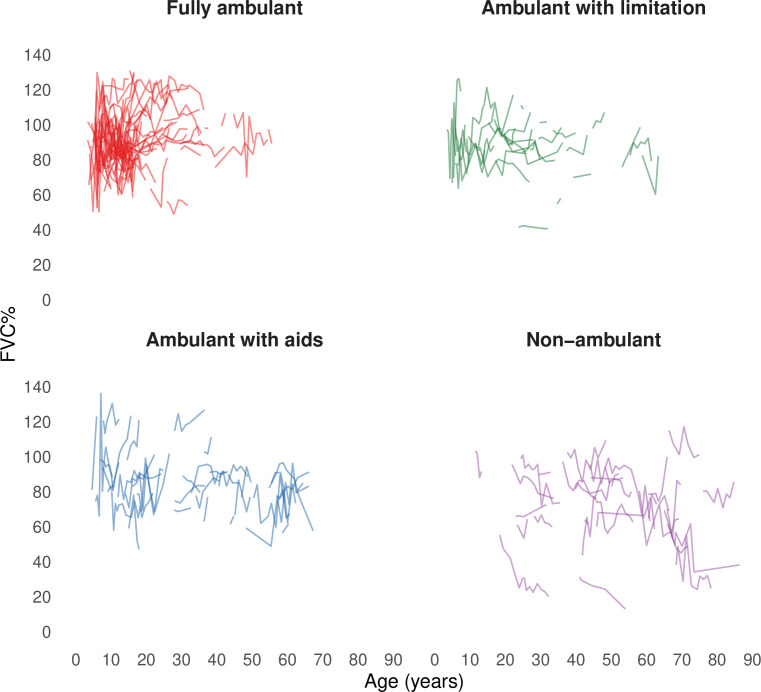
Spaghetti plot of individual FVC% trajectories (raw data) by age across disease motor milestones. FVC%, forced vital capacity percentage of predicted.

To assess FVC% values across disease motor milestones, we analysed 1,146 FVC% measurements from 150 individuals for whom the disease motor milestone at the time of assessments could be established (figure 3). Measurements from the same subjects were included in more than one category if they transitioned across disease motor milestones over time.

In the single-variable model, we separately evaluated previously considered predictors (cardiac involvement, *DMD* gene variant subtypes, OSA, asthma, COPD and other severe respiratory comorbidities). None of these reached statistical significance and were therefore excluded from the multi-variable model.

**Figure 4:**
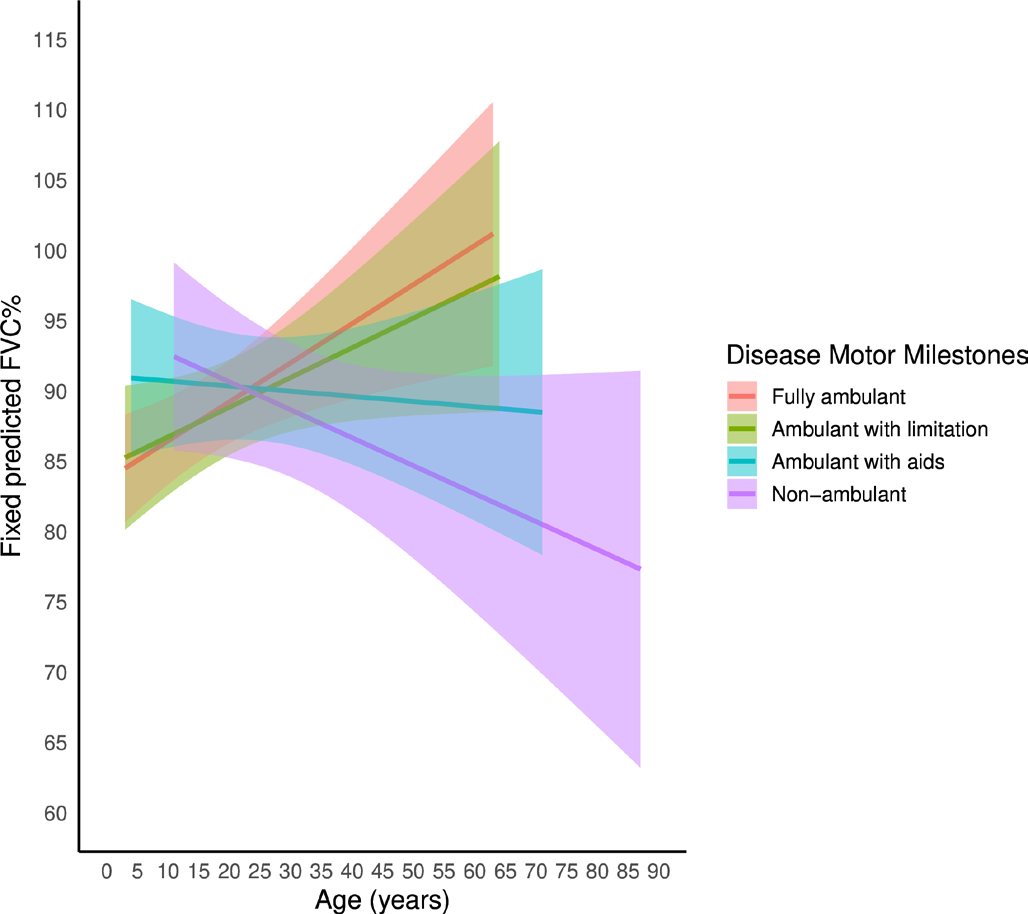
Predicted model curves based on fixed effects of disease motor milestones, with 95% CIs. The curves indicate that respiratory decline appears to begin during the ‘ambulant with aids’ phase, becoming more pronounced in the ‘non-ambulant’ stage. FVC%, forced vital capacity percentage of predicted.

Compared with the fully ambulant group, a significant decline in FVC% was observed in the non-ambulant group (estimate −0.48%/year, p=0.0001) and the ambulant with aids group (estimate −0.31%/year, p=0.001), while the change in the ambulant with limitation group was not statistically significant (estimate −0.07%/year, p=0.42) (table 2). Figure 4 illustrates the predicted model FVC% curves based on fixed effects of disease motor milestones.

**Table 2:** Estimated changes in the forced vital capacity percentage of predicted (FVC%) values for each disease motor milestone by year

Disease motor milestone	Patients included (n)*	Observations (n)*	Estimated annual change (FVC%)	SE	95% CI	P value
Fully ambulant	90	529	0.278	0.0966	0.0876 to 0.468	0.004
Ambulant with limitation	47	191	−0.0667	0.0827	−0.229 to 0.0955	0.42
Ambulant with aids	47	220	−0.314	0.0998	−0.510 to −0.118	0.002
Non-ambulant	35	206	−0.477	0.125	−0.722 to −0.231	0.0002

The ‘fully ambulant’ group is set as the reference category to calculate the estimates of the other disease motor milestones.

* Measurements from the same subjects were included in more than one category if they transitioned across disease motor milestones over time.

### Correlation between UL motor function (PUL 2.0 entry item) and FVC%

PUL 2.0 entry item scores at the last assessment were available for 88 individuals (mean age 30.9±18 years, range 5.48–80.3). All patients within the fully ambulant group achieved the maximum score (6 out of 6). PUL 2.0 entry item<3, indicative of loss of the hand-to-mouth function, was observed exclusively in the non-ambulant group (online supplemental figure 1).

**Figure 5:**
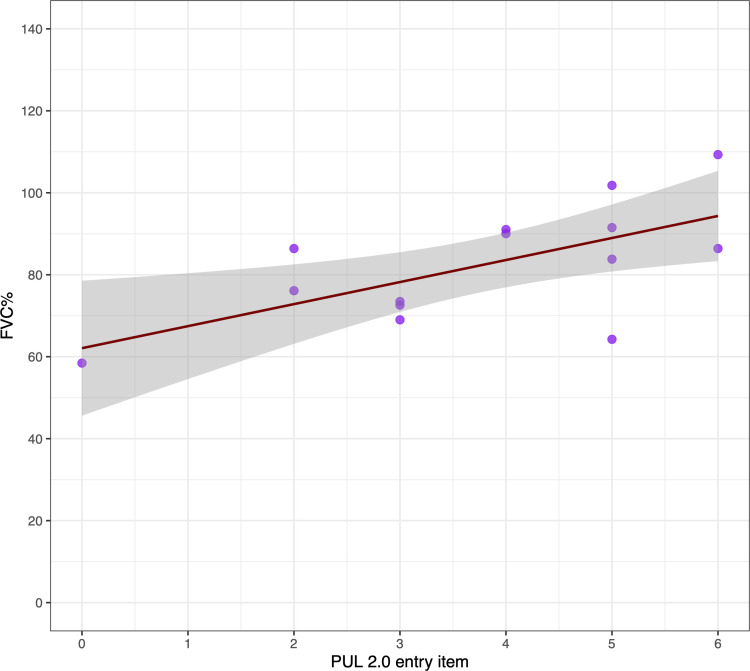
Correlation between FVC% and PUL 2.0 entry item in patients within the non-ambulant disease motor milestone at the last assessment (n=14, mean age 45±18.2 years, range 13.2–80.3; Spearman’s rank correlation coefficient rho=0.60, p=0.02). PUL 2.0 entry item: 0=no useful function of hands; 1=can use hands to hold pen or pick up a token or drive a powered chair; 2=can raise one or two hands to mouth but cannot raise a cup with 200 g weight in it to mouth; 3=can raise standardised plastic cup with 200 g weight in it to mouth using both hands if necessary; 4=can raise both arms simultaneously (elbows to shoulder height-elbow flexed or extended); 5=can raise both arms simultaneously above head only by flexing the elbows; 6=can abduct both arms simultaneously—elbows in full extension until the elbows reach the ears. FVC%, forced vital capacity percentage of predicted.

A positive association was found between FVC% and PUL 2.0 entry item at the last assessment (Spearman’s rank correlation coefficient rho=0.35, p=0.0008; online supplemental figure 2), and it was stronger among non-ambulant individuals (rho=0.60, p=0.02; figure 5).

### Respiratory support

In our cohort, no individual required continuous or invasive ventilation. 17 of 152 patients (11.2%) were receiving nocturnal respiratory support. The mean age at initiation was 50.6±16.0 years and the mean FVC% at the time of initiation (defined as the value closest to initiation within ±18 months) was 65.5±31.4%. FVC% at the time of initiation was, on average, lower in patients prescribed bilevel positive airway pressure (BiPAP; 53.5±32.4%) compared with those prescribed continuous positive airway pressure (CPAP; 80.9±27.4%). The indication for BiPAP was most often symptoms of sleep-disordered breathing, corroborated by overnight monitoring, except for two patients who were initiated following an episode of acute ventilatory failure.

Five of nine (55.6%) patients receiving BiPAP and seven of eight patients (87.5%) receiving CPAP had at least one respiratory comorbidity; all patients with respiratory comorbidities in the CPAP group had a diagnosis of OSA.

BMI data at the last assessment were available for four of eight patients receiving CPAP (mean age 48.4±15.6 years, range 32.1–64.1) and for four of nine patients receiving BiPAP (mean age 53.7±21.7 years, range 34.5–80.3). All patients in the CPAP group fell within the overweight (25–29.9 kg/m²) or obesity (≥30 kg/m²) BMI categories.^21^ In contrast, only half of patients in the BiPAP group (2/4) had an elevated BMI, and one patient was classified as underweight (BMI<18.5 kg/m²).

In non-ambulant individuals, age at initiation of respiratory support was strongly correlated with age at LoA (Spearman’s rank correlation coefficient rho=0.95, p=0.001; online supplemental figure 3).

Additional details regarding patients on respiratory support are provided in table 3.

**Table 3:** Clinical characteristics of patients on respiratory support

Total patients on respiratory support (N)	17/152
CPAP (N)	8/17
BiPAP (N)	9/17
Mean age at initiation (respiratory support)*±SD (range)	50.6±16 years (32.4–85.9)
Mean age at initiation (CPAP)†±SD (range)	51.2±11.5 years (32.4–62.1)
Mean age at initiation (BiPAP)±SD (range)	50.1±19.5 years (33.4–85.9)
Mean FVC (L) at initiation (respiratory support)*‡±SD (range)	2.9±1.5 (0.5–5.2)
Mean FVC (%) at initiation (respiratory support)*‡±SD (range)	65.5±31.4 (10.5–111)
Mean FVC (L) at initiation (CPAP)†‡±SD (range)	3.5±1.4 (1.9–5.2)
Mean FVC (%) at initiation (CPAP)†‡±SD (range)	80.9±27.4 (40.6–111)
Mean FVC (L) at initiation (BiPAP)‡±SD (range)	2.4±1.6 (0.5–4.3)
Mean FVC (%) at initiation (BiPAP)‡±SD (range)	53.5±32.4 (10.5–92.7)
Non-ambulant (respiratory support)±SD (range)	10/17
Non-ambulant (CPAP)§ (N)	3/8
Non-ambulant (BiPAP)§ (N)	7/9
Mean age at LoA (CPAP)±SD (range)	50.5±13.5 years (41.5–66)
Mean age at LoA (BiPAP)¶±SD (range)	30.5±13.7 years (18.4–51.1)
Cardiac involvement (CPAP)§ (N)	8/8
Cardiac involvement (BiPAP)§ (N)	7/9
Mean age at detection-cardiac involvement (CPAP)±SD (range)	44.1±14.7 years (17.6–63)
Mean age at detection-cardiac involvement (BiPAP)±SD (range)	33.4±19.4 years (18–69.4)
Respiratory comorbidities (CPAP)§ (N)	7/8 (7 OSA, 2 asthma, 2 COPD, 2 ‘other’)
Respiratory comorbidities (BiPAP)§ (N)	5/9 (2 OSA, 2 asthma, 3 COPD, 1 ‘other’)

* Information available for 16/17 patients.

† Information available for 7/8 patients.

‡ FVC (L and %): closest value to initiation of respiratory support (±18 months).

§ At the last available assessment.

¶ Information available for 5/7 patients.

BiPAP, bilevel positive airway pressure; COPD, chronic obstructive pulmonary disease; CPAP, continuous positive airway pressure; FVC, forced vital capacity; LoA, loss of ambulation; OSA, obstructive sleep apnoea.

### Diaphragmatic weakness

A total of 387 FVC% measurements in the lying position were available across 109 patients. Among these, 56 evaluations (14.5%) showed a >10% reduction compared with the sitting FVC%, potentially indicating diaphragmatic dysfunction.^22^ These differences were observed in 40 individuals (36.7%) with at least one such occurrence. However, consistent evidence of diaphragmatic weakness, defined as at least two consecutive evaluations with a >10% reduction, including the most recent assessment, was identified in only three patients. All three had documented cardiac involvement (mean age at detection: 24.2±4 years, range 20–28) and no respiratory comorbidities. Only one of them was non-ambulant and was on nocturnal BiPAP. We did not routinely measure maximum inspiratory and expiratory pressures.

### Peak cough flow

A total of 296 PCF measurements (online supplemental figure 4) were obtained from 91 individuals (mean 3.25±2.28 evaluations per patient, range 1–10). Restricting the analysis to assessments performed after the age of 18 years, only 28 values from 13 patients fell below 270 L/min, which was used as a threshold to define adequate cough.^23^ Of these, only two patients showed persistent reduction, defined as at least two consecutive values below threshold, including the most recent assessment. They both had cardiac involvement, but none had respiratory comorbidities.

## Discussion

This is the largest study to date describing respiratory function changes over time in BMD. The age distribution of our cohort is highly representative of BMD, with assessments spanning from early paediatric stages to late elderly phases.

Respiratory function was generally preserved in the overall cohort, with a mild but significant FVC% annual decline observed after 18 years of age. However, substantial inter-patient variability in trajectories was evident, prompting exploration of clinical factors to explain these differences.

Motor function has been associated with respiratory outcomes in BMD, with LoA representing a major disease motor milestone that typically precedes a more rapid decline.^610^,^12^ This relationship was largely confirmed in our study, and discrepancies in previous multicentric cohorts may reflect differences in cohort composition and the variability of motor function assessments across centres.^9^

When examining additional disease motor milestones, annual FVC% estimates showed an apparent increase among fully ambulant individuals, likely driven by the high representation of paediatric patients within this group. This finding may reflect age-related improvements in cooperation and effort during spirometry in children.

LoA proved to be a robust predictor of poorer respiratory outcomes, with non-ambulant patients exhibiting significantly lower mean FVC% values and a more rapid decline over time. Interestingly, our data suggest that decline may begin during the ‘ambulant with aids’ phase, underscoring the need for more vigilant respiratory monitoring during this transitional period.

However, even within the non-ambulant subgroup, respiratory trajectories were heterogeneous.

To better understand this variability, we examined additional clinical aspects which might serve as predictors. UL motor performance, as assessed by the PUL 2.0 entry item, showed a significant correlation with FVC%, particularly in non-ambulant individuals, suggesting it may act as a surrogate marker of respiratory involvement in more advanced disease stages. This finding could support the prediction of respiratory impairment, particularly in patients unable to perform reliable spirometry.^24^

Cardiac involvement emerged as a possible independent predictor of steeper decline in FVC% and FEV_1_% values. However, this observation may be driven by individuals with more advanced cardiomyopathy, for whom severely impaired cardiac function and chamber dilation may contribute to a restrictive respiratory pattern.^25 26^ Nonetheless, this observation remains uncertain and highlights the need for validation in larger cohorts that incorporate longitudinal cardiac assessments and apply standardised definitions of cardiomyopathy.

*DMD* gene variant subtypes did not have a significant impact on FVC% changes. This could be attributed to the predominance of exon 45–47 deletions in our cohort and the insufficient statistical power to detect effects from less common variants. Interestingly, model predictions based on FEV_1_% trajectories suggest that individuals with deletions spanning exons 45–53 may experience more favourable respiratory outcomes. Nevertheless, the small number of patients in this subgroup precludes definitive interpretations.

COPD and other severe respiratory conditions were associated with significantly poorer respiratory function, as expected.^27^ Although this consideration may seem intuitive, it remains clinically relevant within the spectrum of dystrophinopathies. Unlike individuals with DMD,^28^ patients with BMD often reach late adulthood and older age, thereby becoming susceptible to common age-related respiratory comorbidities. Their identification is essential to improve the interpretation of respiratory decline and to optimise clinical decision-making around monitoring and intervention. This observation also highlights the potential value of educational strategies, for example, discouraging smoking, to help reduce the risk of accelerated respiratory decline beyond that attributable to the underlying muscle condition alone.

Indicators of diaphragmatic weakness and PCF were rarely impaired, suggesting that these assessments need not be used routinely in clinic, but rather reserved for cases with specific clinical concerns, such as symptoms suggestive of nocturnal hypoventilation and/or frequent respiratory tract infections.^29^ The need for respiratory support was also uncommon. Available BMI values at the last assessment for patients receiving respiratory support were often in the overweight or obesity ranges, particularly among those receiving CPAP, supporting a possible association between BMI and respiratory function in BMD.^12^ As expected, patients receiving CPAP had, on average, preserved FVC values but were frequently diagnosed with OSA, suggesting that CPAP initiation was more commonly driven by this respiratory comorbidity than BMD-related restrictive respiratory impairment.

This study has limitations, most notably its retrospective design, which may have resulted in missing data. The small number of patients in some analyses may limit their interpretability. Furthermore, spirometry testing carries intrinsic limitations due to variability in compliance and effort, especially in paediatric patients.

Nonetheless, our study has several important strengths. Despite the rarity of BMD, the relatively large sample size and number of longitudinal assessments, the highly specialised clinical setting in which patients were managed, and the monocentric design all contributed to a high degree of data consistency, standardised application of clinical definitions and overall methodological robustness. Our stringent inclusion criteria^6^ ensured accurate selection across the broad spectrum of BMD, from mildly affected individuals to those with more severe phenotypes.^30^

From a care provision standpoint, in children with BMD, respiratory dysfunction appears uncommon, suggesting that routine monitoring may be deferred until adulthood, in contrast with care recommendations for DMD.^16^ Non-ambulant patients warrant more frequent assessments, with the need for assistive walking devices representing a crucial transitional stage for closer respiratory monitoring. Simple UL motor function assessments, without the requirement of specific equipment or patient cooperation, can help identify non-ambulant individuals at higher risk of severe respiratory impairment, particularly when compliance with spirometry is limited.^16 24^

Similarly, severe cardiac involvement may be associated with poorer FVC% outcomes, suggesting that patients with cardiomyopathy may benefit from closer respiratory surveillance.

From a clinical trial perspective, respiratory function measures may serve as reliable parameters for safety monitoring. However, their use as efficacy endpoints appears limited due to the typically slow rate of decline, particularly in ambulant young adults who are the main target population of ongoing trials in BMD.

These findings provide meaningful insight that can help inform international care guidelines and expand knowledge of the natural history of BMD, thereby supporting the interpretation and design of current and future clinical trials.

## Supplementary material

10.1136/jnnp-2025-337953 online supplemental file 1

## Data Availability

Data are available upon reasonable request.

## References

[1] Flanigan KM (2014). Duchenne and Becker muscular dystrophies. Neurol Clin.

[2] Mah JK, Korngut L, Dykeman J (2014). A systematic review and meta-analysis on the epidemiology of Duchenne and Becker muscular dystrophy. Neuromuscul Disord.

[3] Monaco AP, Bertelson CJ, Liechti-Gallati S (1988). An explanation for the phenotypic differences between patients bearing partial deletions of the DMD locus. Genomics.

[4] Neri M, Rossi R, Trabanelli C (2020). The Genetic Landscape of Dystrophin Mutations in Italy: A Nationwide Study. Front Genet.

[5] Bushby KMD, Gardner-Medwin D (1993). The clinical, genetic and dystrophin characteristics of Becker muscular dystrophy. J Neurol.

[6] Riguzzi P, Borland H, James MK (2025). Characterisation of a large, single-centre cohort of patients with Becker muscular dystrophy to inform standardised care guidelines. J Neurol.

[7] Kaspar RW, Allen HD, Ray WC (2009). Analysis of dystrophin deletion mutations predicts age of cardiomyopathy onset in becker muscular dystrophy. Circ Cardiovasc Genet.

[8] Finsterer J, Stöllberger C (2008). Cardiac involvement in Becker muscular dystrophy. Can J Cardiol.

[9] Nakamura A, Matsumura T, Ogata K (2023). Natural history of Becker muscular dystrophy: a multicenter study of 225 patients. Ann Clin Transl Neurol.

[10] Gorgoglione D, Sabbatini D, Riguzzi P (2025). Natural history of Becker muscular dystrophy: DMD gene mutations predict clinical severity. Brain (Bacau).

[11] Mori-Yoshimura M, Oya Y, Komaki H (2020). Respiratory Dysfunction in Becker Muscular Dystrophy Patients: A Case Series and Autopsy Report. J Neuromuscul Dis.

[12] De Wel B, Willaert S, Nadaj-Pakleza A (2021). Respiratory decline in adult patients with Becker muscular dystrophy: A longitudinal study. Neuromuscul Disord.

[13] Schiava M, Lofra RM, Bourke JP (2024). Functional abilities, respiratory and cardiac function in a large cohort of adults with Duchenne muscular dystrophy treated with glucocorticoids. Eur J Neurol.

[14] Muni-Lofra R, Juanola-Mayos E, Schiava M (2023). Longitudinal Analysis of Respiratory Function of Different Types of Limb Girdle Muscular Dystrophies Reveals Independent Trajectories. Neurol Genet.

[15] Magot A, Wahbi K, Leturcq F (2023). Diagnosis and management of Becker muscular dystrophy: the French guidelines. J Neurol.

[16] Childs A-M, Turner C, Astin R (2024). Development of respiratory care guidelines for Duchenne muscular dystrophy in the UK: key recommendations for clinical practice. Thorax.

[17] Graham BL, Steenbruggen I, Miller MR (2019). Standardization of Spirometry 2019 Update. An Official American Thoracic Society and European Respiratory Society Technical Statement. Am J Respir Crit Care Med.

[18] Bowerman C, Bhakta NR, Brazzale D (2023). A Race-neutral Approach to the Interpretation of Lung Function Measurements. Am J Respir Crit Care Med.

[19] Quanjer PH, Stanojevic S, Cole TJ (2012). Multi-ethnic reference values for spirometry for the 3-95-yr age range: the global lung function 2012 equations. Eur Respir J.

[20] Mayhew AG, Coratti G, Mazzone ES (2020). Performance of Upper Limb module for Duchenne muscular dystrophy. Dev Med Child Neurol.

[21] Nuttall FQ (2015). Body Mass Index: Obesity, BMI, and Health: A Critical Review. Nutr Today.

[22] American Thoracic Society/European Respiratory Society (2002). ATS/ERS Statement on Respiratory Muscle Testing. Am J Respir Crit Care Med.

[23] Brennan M, McDonnell MJ, Duignan N (2022). The use of cough peak flow in the assessment of respiratory function in clinical practice- A narrative literature review. Respir Med.

[24] Borland H, Moore U, Dressman HG (2024). Performance of upper limb entry item to predict forced vital capacity in dysferlin-deficient limb girdle muscular dystrophy. Neuromuscul Disord.

[25] Agostoni P, Cattadori G, Guazzi M (2000). Cardiomegaly as a possible cause of lung dysfunction in patients with heart failure. Am Heart J.

[26] Olson TP, Beck KC, Johnson BD (2007). Pulmonary function changes associated with cardiomegaly in chronic heart failure. J Card Fail.

[27] Cukic V, Lovre V, Ustamujic A (2013). The Changes of Pulmonary Function in COPD During Four-Year Period. Mater Sociomed.

[28] Broomfield J, Hill M, Guglieri M (2021). Life Expectancy in Duchenne Muscular Dystrophy: Reproduced Individual Patient Data Meta-analysis. Neurology (ECronicon).

[29] Chatwin M, Toussaint M, Gonçalves MR (2018). Airway clearance techniques in neuromuscular disorders: A state of the art review. Respir Med.

[30] Hoffman EP (2025). The Persistence of Duchenne vs Becker Muscular Dystrophies: *Vive la Difference*?. Neurol Genet.

