# Supplementary material for: Respiratory function in Becker muscular dystrophy: a comprehensive longitudinal study
Source: J Neurol Neurosurg Psychiatry. 2026 Feb 27;97(5):e337953. doi: 10.1136/jnnp-2025-337953 (PMC13151537; doi:10.1136/jnnp-2025-337953)
Supplement: online supplemental file 1 [file jnnp-97-5-s001.pdf]

Definitions:

Disease motor milestones:

1) Fully ambulant: individuals able to walk independently, without the use of aids, and with no limitations in walking distance.
2) Ambulant with limitation: individuals able to walk independently, without the use of aids, but with significantly restricted walking distances.
3) Ambulant with aids: individuals able to walk only with the assistance of crutches, a walking stick, assistance of another person, and individuals requiring part-time use of a wheelchair.
4) Non-ambulant: individuals requiring a wheelchair full-time.

Cardiac involvement:

Cardiac involvement was defined based on echocardiography finding of left ventricular ejection fraction less than or equal to 50%, and/or fractional shortening less than 27%, and/or left ventricular segmental hypokinesia, and/or evidence of a non-ischaemic distribution of myocardial fibrosis on cardiac MRI.

Supplementary Table 1. *DMD* gene variants, "mild" and "other" subtypes

***DMD* gene subtype**	***DMD* gene variant**	**Number**
Mild	Deletion of exons 45-55	4
Mild	Deletion of exons 48-51	3
Mild	Deletion of exons 49-51	<3
Mild	Deletion of exon 48	<3
Other	Duplication of exons 13-16	7
Other	Deletion of exons 45-49	6
Other	c.1812+1G>A	4
Other	Duplication of exons 10-18	3
Other	Duplication of exons 3-4	3
Other	c.831G>A (p.Gln277Gln)	<3
Other	Deletion of exon 3	<3
Other	Deletion of exons 78-79	<3
Other	Duplication of exons 14-27	<3
Other	Duplication of exons 52-55	<3
Other	c.10454delT (p.Leu3485fs)	<3
Other	c.4071G>C (p.Glu1357Asp)	<3
Other	Deletion of exons 2-7	<3
Other	Deletion of exons 3-5	<3
Other	Deletion of exons 43-44	<3
Other	Deletion of exons 48-49	<3
Other	Deletion of exons 8-19	<3
Other	Deletion of exons 9-41	<3
Other	Duplication of exons 13-29	<3
Other	c.3432+2036A>G	<3
Other	c.1602G>C p.(p.Lys534Asn)	<3
Other	c.31+1G>T1	<3
Other	c.676-678delAAG (p.Lys226del)	<3
Other	c.9563+5G>T	<3

Supplementary Table 2. Longitudinal changes in FEV1% estimated with the linear mixed-effects model

**Stratification**	**Estimated annual change (FEV1%)**	**Standard Error**	***p* value**
Overall cohort	-0.20%/year	1.88	0.016
<18 years of age	+0.53%/year	0.21	0.014
≥18 years of age	-0.54%/year	0.096	<0.0001

Supplementary Table 3. Single-variable model assessing predictors of FEV1% changes

**Predictors (single-variable model)**	**Estimated annual change (FEV1%)**	**Standard Error**	***p* value**
LoA	-0.56%/year	0.18	0.002
Cardiac involvement	-0.37%/year	0.17	0.03
COPD	-1.10%/year	0.35	0.002
Other severe respiratory comorbidities	-1.32%/year	0.43	0.003
Deletion of exons 45-53	+0.69%/year	0.34	0.046

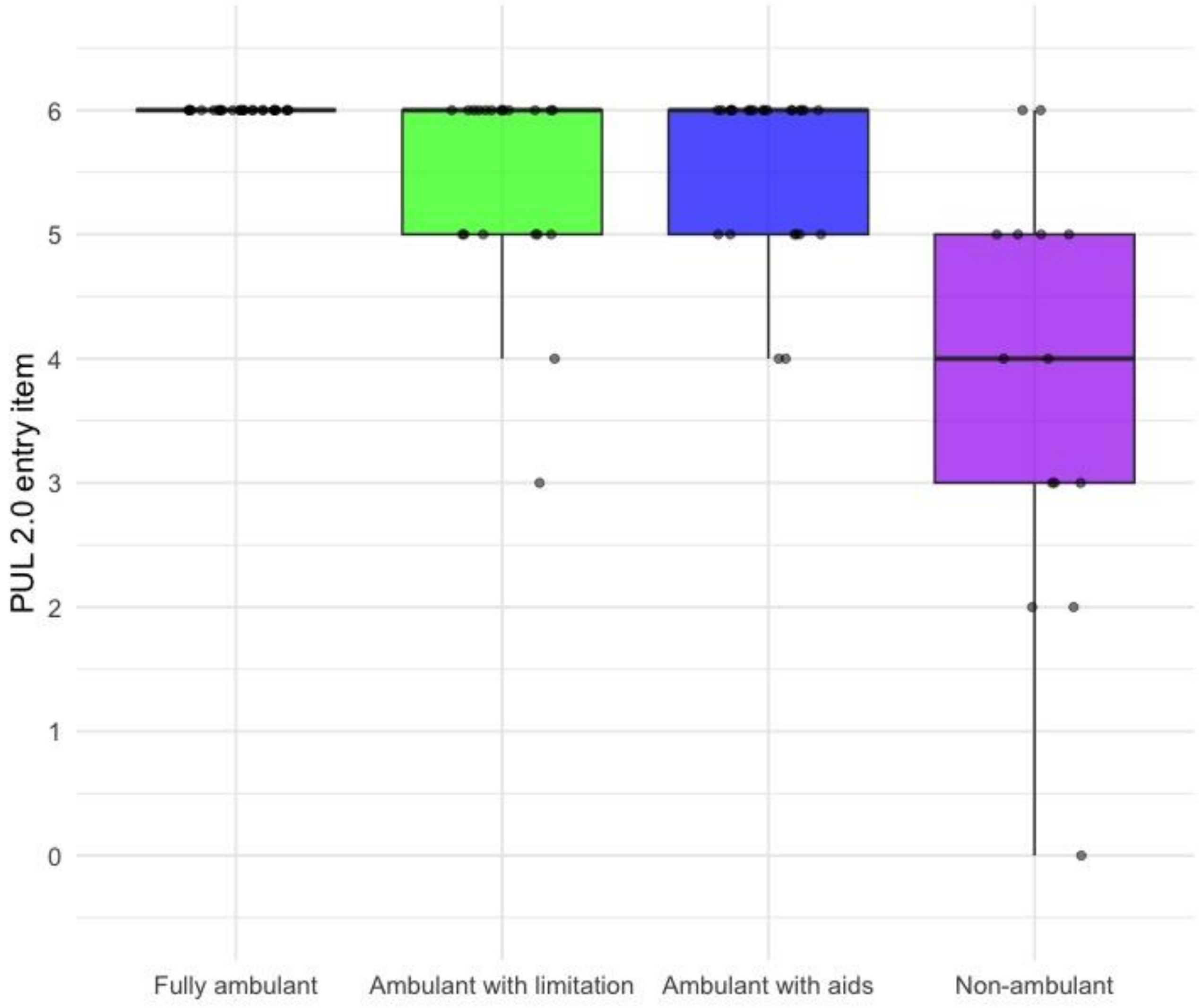

Supplementary Figure 1. Boxplot representing PUL 2.0 entry item scores (median and interquartile range) across disease motor milestones at the last assessment

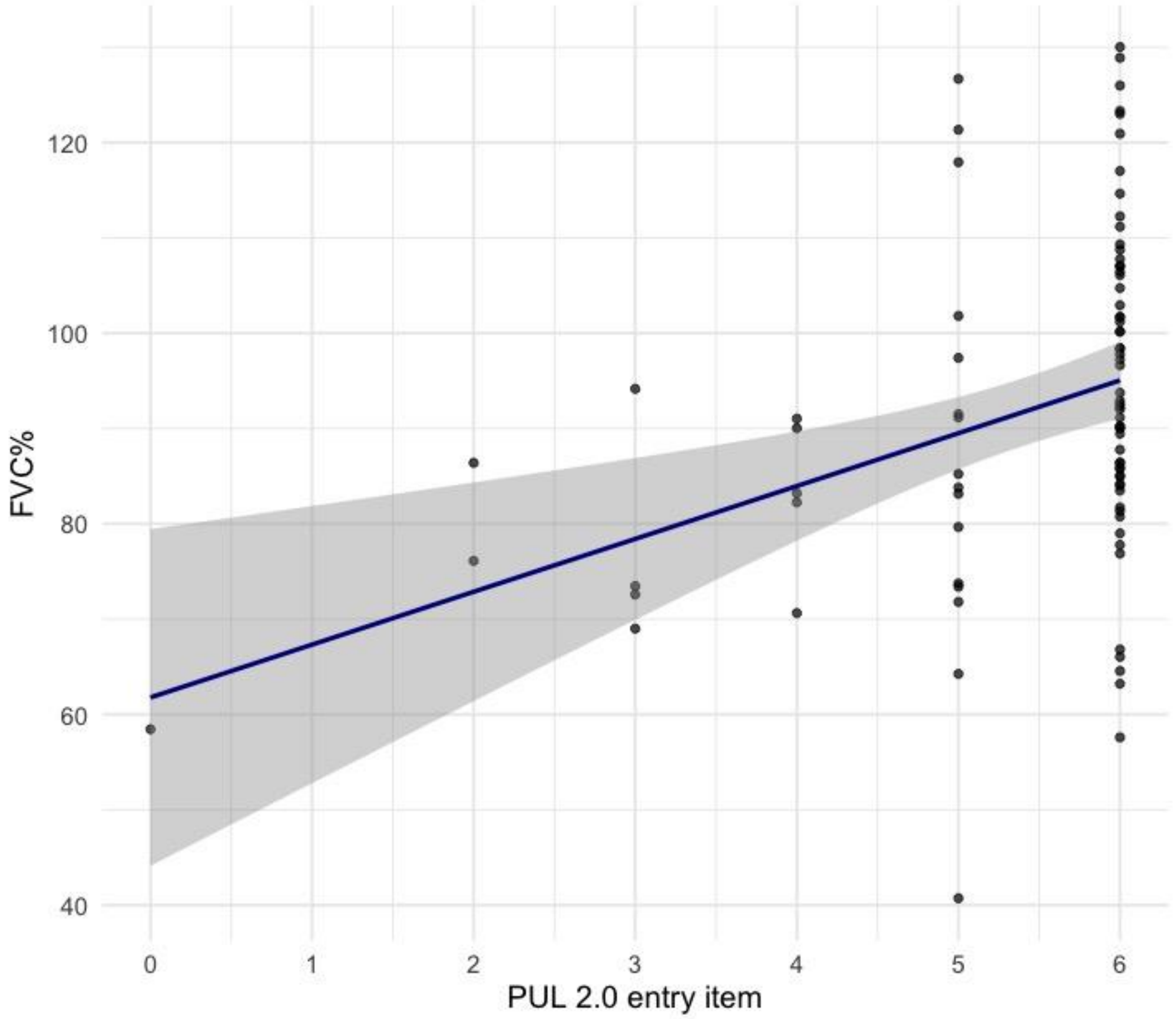

Supplementary Figure 2. Correlation between FVC% and PUL 2.0 entry item scores (whole cohort, n=88) at the last assessment (Spearman's rank correlation coefficient rho= 0.35, *p*=0.0008)

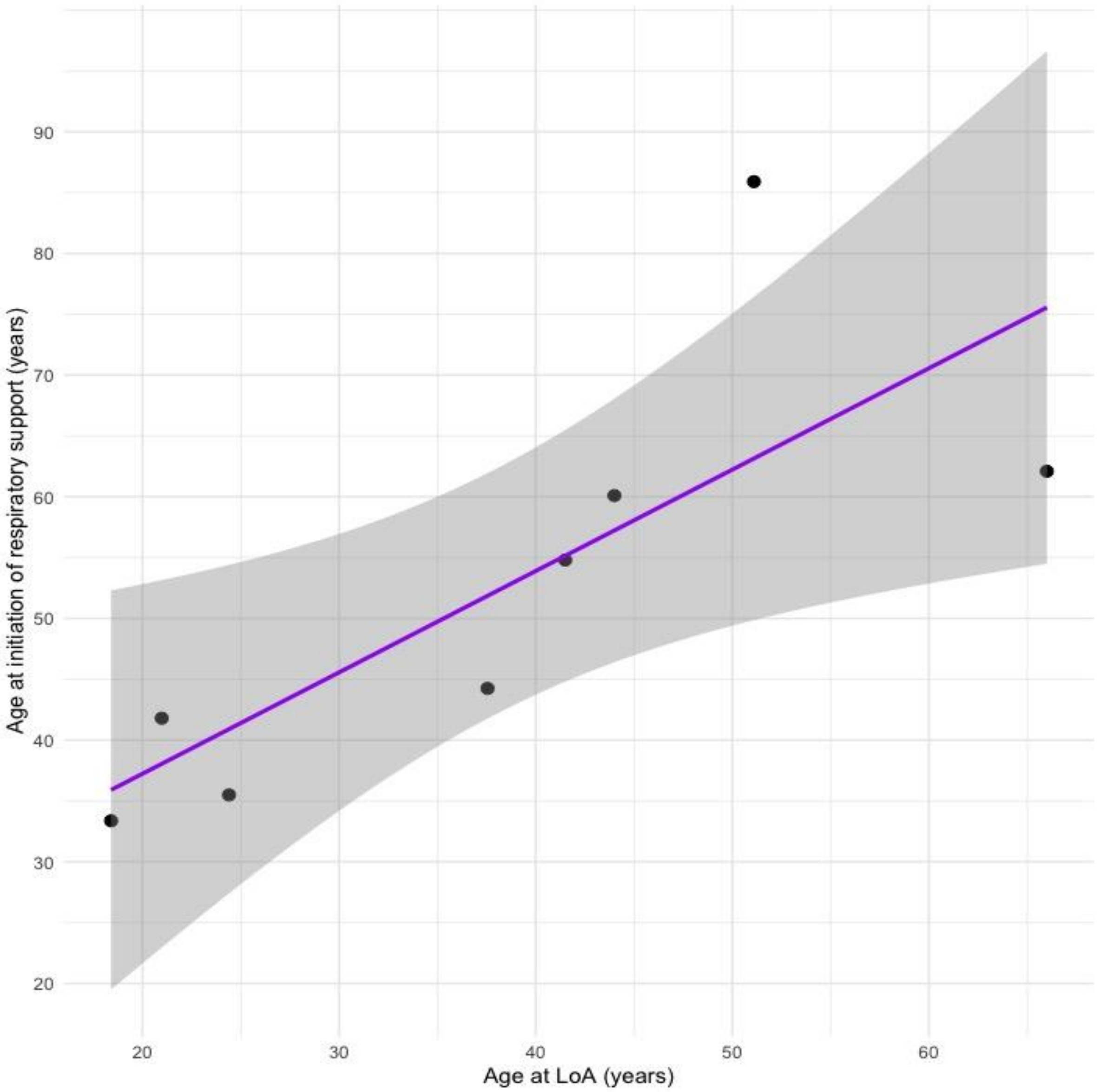

Supplementary Figure 3. Correlation between age at loss of ambulation (LoA) and age at initiation of respiratory support (Spearman's rank correlation coefficient rho=0.95, *p*=0.001)

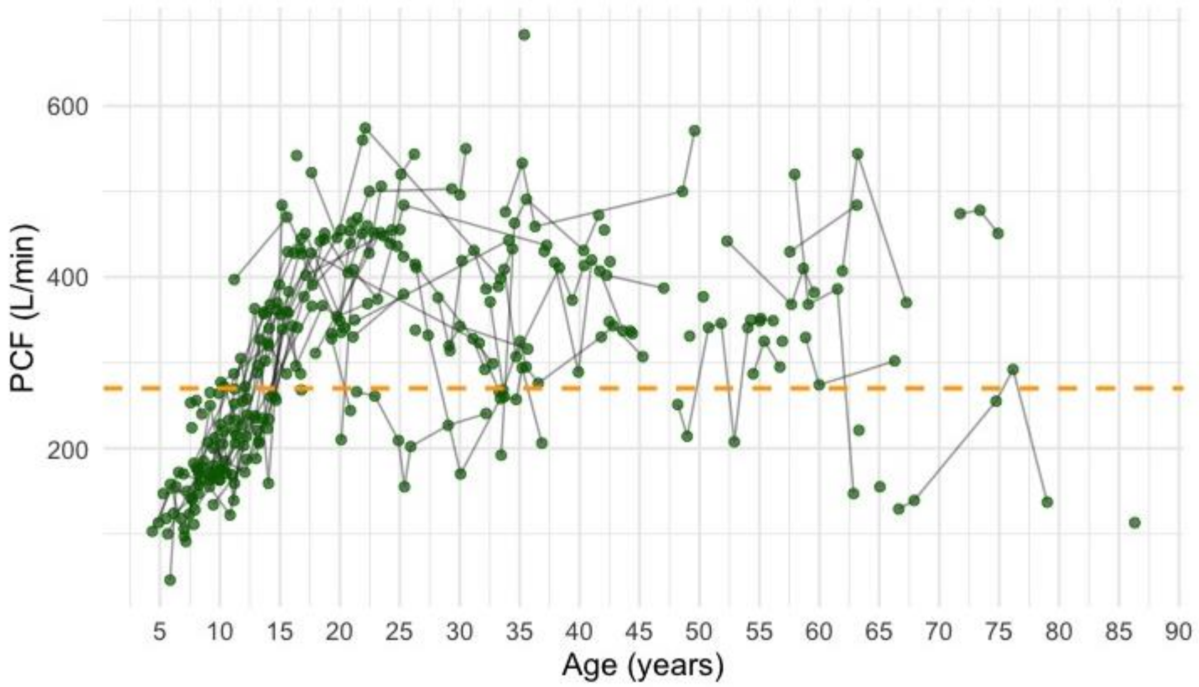

Supplementary Figure 4. Spaghetti plot representing peak cough flow (PCF) trajectories (raw data). The dashed yellow line represents the 270 L/min threshold
